# The Human SLC1A5 (ASCT2) Amino Acid Transporter: From Function to Structure and Role in Cell Biology

**DOI:** 10.3389/fcell.2018.00096

**Published:** 2018-09-04

**Authors:** Mariafrancesca Scalise, Lorena Pochini, Lara Console, Maria A. Losso, Cesare Indiveri

**Affiliations:** ^1^Department DiBEST (Biologia, Ecologia, Scienze Della Terra) Unit of Biochemistry and Molecular Biotechnology, University of Calabria, Cosenza, Italy; ^2^CNR Institute of Biomembranes, Bioenergetics and Molecular Biotechnology, Bari, Italy

**Keywords:** ASCT2, SLC1 family, glutamine, drug design, proteoliposomes, molecular docking

## Abstract

SLC1A5, known as ASCT2, is a neutral amino acid transporter belonging to the SLC1 family and localized in the plasma membrane of several body districts. ASCT2 is an acronym standing for Alanine, Serine, Cysteine Transporter 2 even if the preferred substrate is the conditionally essential amino acid glutamine, with cysteine being a modulator and not a substrate. The studies around amino acid transport in cells and tissues began in the ‘60s by using radiolabeled compounds and competition assays. After identification of murine and human genes, the function of the coded protein has been studied in cell system and in proteoliposomes revealing that this transporter is a Na^+^ dependent antiporter of neutral amino acids, some of which are only inwardly transported and others are bi-directionally exchanged. The functional asymmetry merged with the kinetic asymmetry in line with the physiological role of amino acid pool harmonization. An intriguing function has been described for ASCT2 that is exploited as a receptor by a group of retroviruses to infect human cells. Interactions with scaffold proteins and post-translational modifications regulate ASCT2 stability, trafficking and transport activity. Two asparagine residues, namely N163 and N212, are the sites of glycosylation that is responsible for the definitive localization into the plasma membrane. ASCT2 expression increases in highly proliferative cells such as inflammatory and stem cells to fulfill the augmented glutamine demand. Interestingly, for the same reason, the expression of ASCT2 is greatly enhanced in many human cancers. This finding has generated interest in its candidacy as a pharmacological target for new anticancer drugs. The recently solved 3D structure of ASCT2 will aid in the rational design of such therapeutic compounds.

## Introduction

Amino acids are fundamental metabolites because they are involved in many processes ranging from protein synthesis to energy production and signaling. Intracellular and, hence, plasmatic concentrations of proteogenic amino acids are dynamic and depend on a concerted action of two tightly regulated cell pathways, namely mTOR and GCN2 cascades (Laplante and Sabatini, 2012; B'Chir et al., 2013). These pathways sense the availability of intracellular amino acids and regulate the response to changes of their levels according to cell needs. Alterations of intracellular amino acid content is threatening for cell survival; therefore, a complex network of enzymes and membrane transporters is involved in maintenance of homeostasis and homeorhesis, i.e., flow of amino acids, which is considered even more important than keeping constant concentrations *per se* (Christensen, 1990; Taylor, 2014; Carroll et al., 2015).

### Membrane transporters and metabolism of amino acids

**Figure 1:**
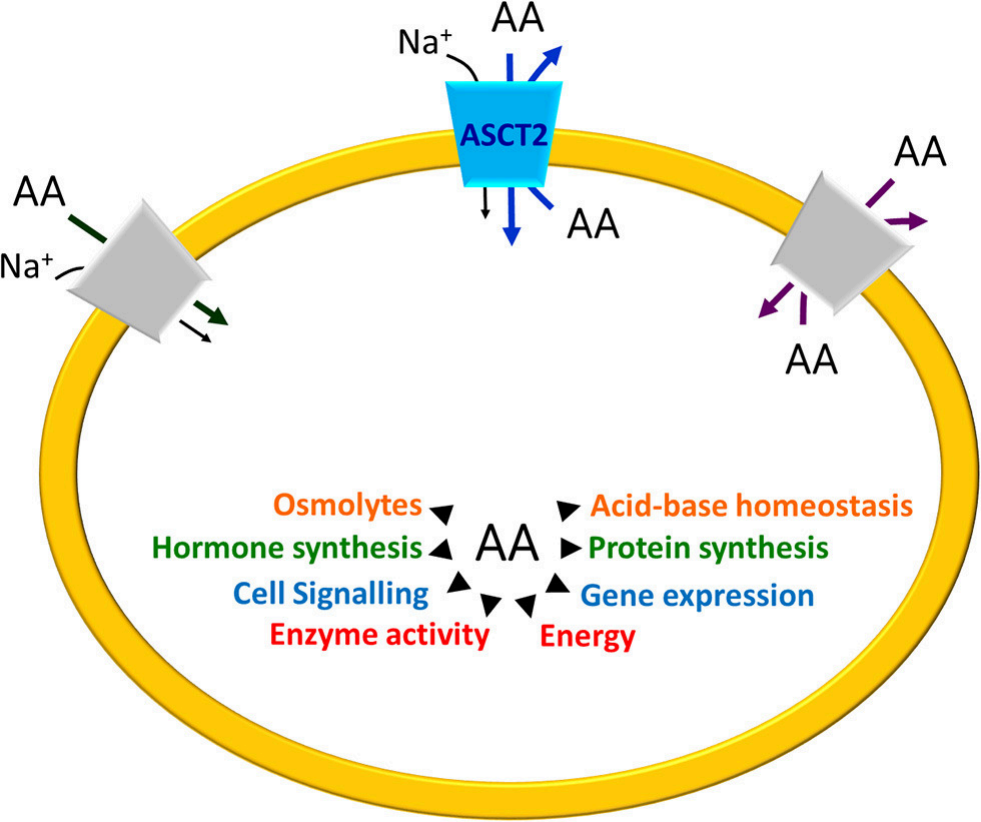
Schematic representation of Amino Acid (AA) roles in cells. The arrows indicate the main processes in which AA are involved. On the plasma membrane of a generic cell, the different transport mechanisms of amino acid transporters are depicted: Na^+^– cotransport (dark gray), amino acid antiport (light gray) and, in the middle, ASCT2 (blue) that is a Na^+^ dependent antiporter with features overlapping both the co-transporters and the antiporters.

Specific enzymes are responsible for reactions of synthesis or degradation of amino acids with productions of secondary metabolites with different fate (Figure 1). Two important examples are (i) the anaplerotic reactions such as the conversion of glutamate to α-ketoglutarate for TCA cycle fueling and (ii) production of gasotransmitters, NO and H_2_S from arginine and cysteine, respectively (Wu, 2009). In this frame, membrane transporters are responsible for absorption, distribution and excretion of amino acids and their derivatives supporting metabolism. Due to the compartmentalization of biochemical pathways in eukaryotes, transporters are necessary either on the plasma membrane and on membranes of intracellular organelles. The flux of proteogenic amino acids is guaranteed in cells by different families of transporters belonging to SLC classification. At least SLC1, 3, 6, 7, 32, 36, 38, and 43 play this function and are characterized by different transport mechanisms, specificities, cellular, and sub-cellular localization and regulations (Hediger et al., 2013). A common characteristic for amino acid transporters is their quite broad specificity and redundancy probably linked to the importance played by these nutrients in cells that employ different mechanisms to guarantee their absorption and balance (Figure 1).

### The conditionally essential amino acid glutamine

The sharp classification in essential and non-essential amino acids has been reconsidered over the years with particular reference to a specific amino acid, i.e., glutamine. It is now well-assessed that glutamine is, indeed, a conditionally essential amino acid because, in some specific physio-pathological situations, the biosynthesis is not sufficient to adequately respond to cell demands, as it was initially observed long ago in cell cultures (Eagle, 1955). Glutamine is a precursor for biosynthesis of proteins, nucleotides and amino sugars; it has a role as a scavenger of NH_3_ in the urea cycle and in plasma for detoxification. Furthermore, the carbon skeleton of glutamine is used for energy production in mitochondrial TCA. The need of glutamine is enhanced in situations of high proliferation rate, such as in inflammatory and stem cells, when all biosynthetic and metabolic demands dramatically increase (Newsholme et al., 2003; Curi et al., 2005; Pochini et al., 2014). Therefore, glutamine concentration in plasma and in cells is kept constant by a conspicuous number of membrane transporters whose study has increased given also the potential outcome for human health.

### The glutamine transporter SLC1A5

Among these proteins, one of the most studied is the plasma membrane transporter SLC1A5, known as ASCT2. The SLC1 family encompasses other six members, besides ASCT2: the neutral amino acid transporter ASCT1 (SLC1A4) and five high affinity glutamate transporters (SLC1A1-3 and SLC1A6-7) (Kanai et al., 2013). In humans, the glutamate transporters share 44–55% sequence identity and the neutral amino acid transporters exhibit 57% identity with each other. The two subgroups of transporters have different tissue distribution and transport mechanism linked to their substrate specificity. ASCT2 is an acronym standing for Alanine, Serine, Cysteine Transporter 2. Over the years, this historical name revealed misleading because the preferred substrate that underlies the main physiological functions of ASCT2 is glutamine that is exchanged, in a Na^+^ dependent manner, with other neutral amino acids such as serine or asparagine or threonine; furthermore, the role of cysteine as a substrate is questionable (see section ASCT2 Gene Expression). Notwithstanding such discrepancies, the ASCT2 acronym continues to be a custom. The features of a transporter that undoubtedly coincides with ASCT2, have been already described in the pre-genomic era. Later, the genes encoding for ASCT2 have been identified in mouse, rat and humans and the corresponding proteins have been studied using different experimental models (Kekuda et al., 1996, 1997; Utsunomiya-Tate et al., 1996; Torres-Zamorano et al., 1998; Bröer et al., 1999; Oppedisano et al., 2004; Pingitore et al., 2013). The physiological role of ASCT2 lies on its peculiar antiport mode of transport coupled to sodium gradient that is responsible for regulating pools of amino acids in intra and extra cellular spaces (Figure 1).

### The glutamine transporter SLC1A5 and cancer

Besides this function in cell biology, ASCT2 is also relevant for human health due to its involvement in several cancers. The link of ASCT2 with this pathology was systematically postulated in 2005 when an eminent review pointed out that this protein supplies cancer cells with glutamine to satisfy specific metabolic and signaling needs (Fuchs and Bode, 2005) (see section ASCT2 and Glutamine in Cell Metabolism). Then, this feature revealed a hallmark of human cancers and, consequently, ASCT2 has been elected as a pharmacological target for specifically blocking cancer cells growth and development (Ganapathy et al., 2009; Bhutia and Ganapathy, 2016; Bröer, 2018). Therefore, big efforts are underway to design new drugs; however, the lack of the 3D structure hampered the achievement of this effort, as in the case of other membrane transporters. Indeed, homology structural models for docking were obtained using as templates the GLTph structure or the more recent human SLC1A3 (EAAT1) structure (Yernool et al., 2004; Canul-Tec et al., 2017). In this scenario, an important outcome is the 3D structure of the human ASCT2 that has been recently solved opening new perspective in pharmacological studies (Garaeva et al., 2018). The fundamental role of ASCT2 transporter in cell biology and in human health is testified by the over 100 articles published in the last 10 years (using as keywords ASCT2 or SLC1A5 in title/abstract field on https://www.ncbi.nlm.nih.gov/pubmed) dealing with this protein. Therefore, the present review aims to summarize the information collected on ASCT2 giving an overview of the main findings with a focus on the pathophysiological aspects.

## ASCT2 gene expression

**Figure 2:**
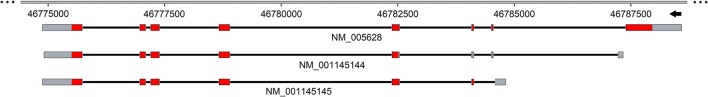
Schematic representation of human SLC1A5 gene. Exons and introns are depicted with solid boxes and black straight lines, respectively. UTR sequence is highlighted in gray while the coding region in red. Genbank accession number is reported below each of the transcripts. The scheme is realized using SnapGene tool.

The SLC1A5 gene is located at 19q13.3 and contains 8 exons. In NCBI and Ensemble database, 3 transcripts are reported for the human gene deriving from different translation start. The first variant NM_005628 represents the longest transcript, constituted by 2882 nucleotides and 8 exons. This transcript encodes a peptide of 541 amino acids. The second variant NM_001145144 is constituted by 1750 nucleotides and differs in the 5′ UTR from the variant NM_005628. In NM_001145144 the translation starts downstream the third exon generating a shorter peptide of 313 aa. The third isoform NM_001145145 has 1872 nucleotides and lacks the first exon; it presents a different translation start at 5′, coding a peptide of 339 amino acids. A longer transcript, XM_005259167, is reported only in the NCBI database, which has been identified by automated computational analysis (Figure 2).

### Naturally occurring variant of the human SLC1A5 gene

More than 6000 SNP(s), of the SLC1A5 gene, are reported in the dbSNP database (http://www.ncbi.nlm.nih.gov/snp/). Among these 347 SNPs are missense, 13 are non-sense and 12 insert a stop codon. The others are in 3′ or 5′ UTR and introns. Some of the SNPs have been also characterized: the variants rs3027956 and rs11668878 are associated with breast cancer (Savas et al., 2006) and chronic lymphocytic leukemia (Sillé et al., 2012), respectively. The variants rs3027985 and rs1644343 are linked to longevity; in particular, by *in vitro* assays it has been demonstrated that both SNPs positively influence the probability to become long-lived (D'Aquila et al., 2017). Moreover, a region constituted of 907 bp upstream of the ASCT2 gene possesses promoter activity (Bungard and McGivan, 2004).

### Cellular sub-localization and tissue distribution of human ASCT2

The gene is found in 56 different organisms (http://www.ncbi.nlm.nih.gov/gene/6510) and is virtually present in all vertebrates. The better-known orthologous of the human gene are those from rat, mouse and rabbit with the identity of 79, 82, and 85%, respectively. The gene SLC1A5 encodes for a protein, named ASCT2 (aliases: AAAT, ATBO, M7V1, M7VS1, R16, RDRC) with a unique localization at the plasma membrane of cells. The protein is broadly expressed in lung, skeletal muscle, large intestine, kidney, testis, T-cells, brain, and adipose tissue (Kanai et al., 2013; Poffenberger and Jones, 2014). As reported in atlas protein database, ASCT2 expression increases dramatically in cancers: colorectal, prostate, hepatic, lung, breast, cervical, ovarian, renal, and brain cancers reviewed in Scalise et al. (2017). Noteworthy, ASCT2 expression is detected also in cancers deriving from human tissues in which normally the protein is not present. Very recently, it has been reported that ASCT2 has a major role in hematopoietic stem cells lineage specification; blockade or silencing of this transporter triggers a dramatic reduction of glutamine uptake with consequent inhibition of erythroid differentiation (Oburoglu et al., 2014).

## Transport properties

### ASCT2 discrimination from similar transporters

As stated in the introduction, the investigation around ASCT2 transport function started in the ‘60s using brush border vesicles and tumor ascites as the experimental models. Different groups of amino acid transporters, called “systems,” were roughly classified in Na^+^– independent and Na^+^– dependent members (Christensen et al., 1965, 1967). Since its first discovery, a transport system called ASC was included in this second category due to the strong dependence on Na^+^ (Christensen, 1990). At that time, the gene was still unknown and the transport was measured in the brush border of mouse intestine and kidney. ASC was distinguished from system A (Alanine- preferring) and L (Leucine- preferring) according to different sensitivity toward specific inhibitors of system A and L, namely MeAIB and BCH, respectively; in the meanwhile, the same system was also defined B by other authors to distinguish its specificity for neutral amino acids from B^0, +^ that is, on the contrary, specific for both neutral and cationic amino acids [(Bode, 2001; McGivan and Bungard, 2007) and refs herein]. As a matter of curiosity, B^0, +^ corresponds to the actual ATB^0, +^ (SLC6A14) and is, indeed, specific for both neutral and cationic amino acids (Pochini et al., 2014). At that time, the investigation of the transport mechanism was hampered by the intrinsic complexity of the experimental tool as well as by the general lack of knowledge on membrane transport. Notwithstanding, those pioneering studies highlighted the existence of both a “trans-stimulation” phenomenon (Gazzola et al., 1980) and a bidirectional transport of glutamine across rat liver membrane with characteristics that were not attributable to system N, A, or L (Fafournoux et al., 1983). These findings suggested that the system ASC could exhibit an antiport mode of transport for glutamine.

### Cloning and characterization of the SLC1A5 gene

During the 90's, in the course of these intricate studies, genes started to be annotated in mouse, rat and rabbit genomes. Thus, transport proteins encoded by the newly annotated genes, were studied in intact cells or heterologously expressed in *X. laevis* oocytes by RNA injection (Kekuda et al., 1996, 1997; Utsunomiya-Tate et al., 1996; Bröer et al., 1999). For a while, the proteins were named differently, i.e., ASCT2 and ATB^0^, because they were considered two distinct molecular entities. In 2000, it was definitively assessed that ASCT2 and ATB^0^ are the same transport system, orthologues of different species (Broer et al., 2000). Human ASCT2 was also distinguished from human ASCT1 that belongs to the same family and was identified in 1993 (Shafqat et al., 1993). In particular, ASCT1 has a narrow substrate specificity that does not include glutamine and other ASCT2 substrates (Arriza et al., 1993; Shafqat et al., 1993).

### ASCT2 substrates and transport mechanism

Human ASCT2 is an obligatory Na^+^-dependent exchanger of neutral amino acids that does not tolerate substitution of Na^+^ with Li^+^ or K^+^ (Kekuda et al., 1996, 1997; Utsunomiya-Tate et al., 1996; Bröer et al., 1999; Bode, 2001). The antiport mode of transport has been clearly described by following both uptake and efflux of radiolabeled substrates in intact cells and in oocytes expressing the transporter and by inhibition analysis (Kekuda et al., 1996, 1997; Utsunomiya-Tate et al., 1996; Bröer et al., 1999; Bode, 2001). In these conditions, substrate specificity has been suggested: the transporter showed capacity of mediating uptake and efflux of radiolabeled alanine, glutamine and threonine. Inhibition analyses indicated that leucine, threonine, methionine, alanine, glutamine, and glutamate are able to induce efflux of radiolabeled threonine from oocytes expressing human ASCT2, as well as rat ASCT2 (Torres-Zamorano et al., 1998; Broer et al., 2000); thus, according to the obligatory antiport mechanism of ASCT2 they have been also considered substrates of the protein.

#### Species-specific differences in the ASCT2 protein

Using the murine isoform, the Na^+^-dependent uptake of alanine has been also investigated with the patch clamp methodology and, in these electrical measurements, an anion current associated to counter-transport of amino acid has been also observed. The anion current phenomenon showed the following preference: SCN^−^ >> ${\text{NO}}_{3}^{-}$ >I^−^> Cl^−^ (Broer et al., 2000). However, the actual role of this anion leakage is not yet deciphered because the preferred anion SCN^−^ is un-physiological. The kinetic studies on the murine isoform left the issue of Na^+^ cotransport unsolved probably due to intrinsic technical limitations of the intact cell model. In fact, it was not clarified whether Na^+^ is directly involved in the transport reaction as a co-transported ion or simply binds to the transporter stimulating the transport activity. As a consequence, the electrogenicity of transport was not assessed nor excluded (Utsunomiya-Tate et al., 1996; Broer et al., 2000; Zander et al., 2013). Starting from 2004, some more data derived from the rat ASCT2 solubilized from kidney brush border and reconstituted in proteoliposomes with the same orientation as in the cell membrane (right-side-out) (Oppedisano et al., 2004, 2007). With this experimental tool, both uptake and efflux experiments have been conducted and, due to the possibility of controlling the composition of intra and extraliposomal milieu, additional properties of rat ASCT2 were described and some previous doubts were solved. In particular, it was confirmed that glutamine is the preferred substrate of ASCT2 and that the transporter exhibits an asymmetric specificity: alanine, serine, asparagine, threonine, are able to induce both glutamine uptake and efflux, i.e., these substrates could be transported bi-directionally. While valine and cysteine are able to stimulate only glutamine efflux, i.e., they should be only inwardly transported. Moreover, a random simultaneous transport mechanism has been revealed in which the substrates bind to the transporter with no preferential order (Oppedisano et al., 2004, 2007). It has been also definitively demonstrated the Na^+^ is a substrate of ASCT2: in fact, radiolabeled Na^+^ uptake has been measured together with neutral amino acids exchange with a 1:1 stoichiometry giving rise to a peculiar tri-substrate reaction (Oppedisano et al., 2007). On the basis of the expression of ASCT2 in the brain (Utsunomiya-Tate et al., 1996; McGivan and Bungard, 2007), it has been previously hypothesized that the transporter is involved in the glutamine/glutamate cycle and, possibly, in the transport of glutamate. Later on, it has been demonstrated that the rat ASCT2 mediates transport of glutamate in proteoliposomes and that the glutamate/glutamine antiport preferentially occurs at acidic pH on the glutamate side (Oppedisano et al., 2007). These data supported the hypothesis, previously put forward, that ASCT2 could be responsible for glutamate reuptake in astrocytes in exchange for glutamine that, upon entry into neurons through transporter(s) belonging to SNATs (SLC38 family), serves for producing glutamate and GABA used as neurotransmitters (Albrecht, 1989; Leke and Schousboe, 2016; Scalise et al., 2016). It is important to highlight that the same cycle has been proposed to occur also in the placenta to sustain fetus growth and development (Torres-Zamorano et al., 1998; Wu et al., 2015). For the sake of thoroughness, it has to be stressed that all the substrates mentioned so far are L-amino acids; however, ASCT2, together with ASCT1, is also responsible for distribution of D-serine in brain whose concentration needs to be kept constant because its alteration is linked to neurological disorder such as ALS (Lee et al., 2017) (see section Involvement in Human Pathology and Interaction With Drugs).

### Expression and purification of human ASCT2; insights into transport mechanism

The most striking advancements on the ASCT2 knowledge are represented by the studies on the human isoform, which began almost simultaneously to that of murine isoforms (Kekuda et al., 1996; Torres-Zamorano et al., 1998). For a long time, the studies have been conducted with the certainty that the two proteins are virtually identical and, hence, taking results from murine ASCT2 as representative of human ASCT2 behavior. However, it became later evident that identity between rat and human ASCT2 is not as high as for other orthologous transporters (Marin et al., 2003; Pochini et al., 2014). In fact, besides an overall identity of 79% that is somewhat lower than the average identity for other membrane transporters between human and mouse/rat isoforms, in ASCT2 there is a stretch of about 30 amino acids that is profoundly divergent in the different species (Pochini et al., 2014). This peculiarity matches another difference that is the number of cysteine residues: human ASCT2 counts 8 cysteines, while mouse counts 14 and rat 16 cysteines. This characteristic represents an intriguing exception to the evolutionary tendency of increasing the number of cysteine residues in proteins (Jordan et al., 2005). An important advancement in the study of the human isoform was provided by its successful overexpression in the yeast *P. pastoris* (Pingitore et al., 2013). The heterologous protein was purified in large scale and in an active form, which is suitable for both functional (Pingitore et al., 2013) and structural studies (Garaeva et al., 2018). The human protein exhibits kinetic asymmetry, similarly to the rat isoform. External Km for glutamine, threonine, serine, or asparagine is in the micromolar range, much lower than the internal Km for the same substrates, which is in the millimolar range; alanine is only inwardly transported with a Km in the micromolar range (Pingitore et al., 2013; Scalise et al., 2014). The Km values correlate well with the intra and extracellular concentrations of amino acids (Cynober, 2002) further demonstrating the physiological role of ASCT2 as “harmonizer” of amino acid pools (Bröer et al., 2016).

#### The electrogenicity controversy

**Figure 3:**
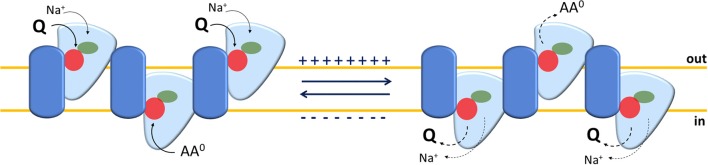
Sketch of the transport mechanism of human ASCT2 homotrimer. The model summarizes the main findings on human ASCT2 collected in both intact cell systems and in proteoliposomes. Each monomer is depicted as a two-domain assembly composed of a fixed part embedded in the plasma membrane (blue) and a mobile part (light blue) that constitutes the elevator domain. The proposed model is a trimer in which the three monomers work independently allowing translocation of glutamine and Na^+^ in an exchange with other neutral amino acids. The red and green circles depict amino acids and Na^+^ binding sites, respectively. The oligomeric structure explains the random simultaneous mechanism in which the transporter exposes outward or inward faces at the same time on each membrane side. In the transport cycle, Na^+^ and Glutamine (Q), bound on the external side (out), are released in the internal side (in) upon moving of the mobile domain; simultaneously, neutral amino acids (AA^0^), bound on internal side (in), are released to the external side (out). The sidedness of membrane potential is indicated. The solid and dotted arrows indicate the loading or the release of the substrate(s) to or from the trimer, respectively.

As stated above, a controversial issue on ASCT2 is the electrical nature of the transport. Indeed, two different hypotheses have been postulated to sustain the existence of an overall electroneutral transport: an exchange of internal Na^+^ or K^+^ with external Na^+^ (Utsunomiya-Tate et al., 1996; Broer et al., 2000). Zander and colleagues proposed that rat ASCT2 mediates a voltage-dependent mechanism based on inwardly directed transport of more than one Na^+^ (Zander et al., 2013). However, the sequence of molecular events underlying this mechanism is not totally supported by experimental data. Information to clarify this issue has been furnished by studies on the human ASCT2 in proteoliposomes conducted in the presence of an artificial membrane potential, mimicking that of the cell membrane, imposed by the ionophore valinomycin. In this experimental setup, the tri-substrate reaction ${\text{Na}}_{\text{ex}}^{+}$-Gln_ex_/ Gln_in_ is stimulated being in good agreement with the inward movement of one Na^+^ during the transport cycle (Figure 3). Interestingly, Na^+^ is needed also in the intraliposomal compartment (intracellular side) but, at a concentration much lower than the external one, corresponding to the physiological intracellular Na^+^ concentration. This effect is based on allosteric regulation of human ASCT2 with no Na^+^ transport (Scalise et al., 2014).

#### The cysteine controversy

The results collected in the first studies on ASCT2, both murine and human, described this protein as responsible also for cysteine transport. However, this information derived mainly from indirect experimental evidences such as inhibition analyses and counter transport assays. In other words, according to the antiport mechanism of ASCT2, an amino acid able to induce efflux of another substrate is considered a substrate, as well. In this case, cysteine is able to induce efflux of radiolabeled substrates such as alanine or glutamine. Noteworthy, experiments performed with a direct procedure, i.e., measuring radiolabeled cysteine transport indicated a very low, if any, transport of this amino acid (Utsunomiya-Tate et al., 1996). Curiously, in the same report on mouse ASCT2, cysteine is described as a strong non-competitive inhibitor of alanine transport (Utsunomiya-Tate et al., 1996). As a matter of history, one of the very first reports dealing with glutamine distribution shows that inhibitory effects of cysteine are probably unspecific due to glutathione depletion and cell membrane damage (Fafournoux et al., 1983). We addressed this issue through a combined experimental approach using both intact human cells and the recombinant human ASCT2 in proteoliposomes. In both systems, cysteine is able to induce efflux of radiolabeled glutamine but it is not taken up, as a substrate, in antiport with the radiolabeled glutamine. Moreover, cysteine is a potent inhibitor of glutamine uptake (Pingitore et al., 2013; Scalise et al., 2015). According to the previous report by Utsonomiya-Tate, the inhibition triggers a mixed or non-competitive behavior even though it is not caused by covalent binding to cysteine residues of the protein (Utsunomiya-Tate et al., 1996; Scalise et al., 2015). The strong and specific binding of cysteine induces an anomalous unidirectional substrate efflux by the protein, which also depends on the presence of extracellular Na^+^ in the transport assay (Scalise et al., 2015). In these conditions, cysteine acts as a regulator of human ASCT2 transport activity. Noteworthy, the previously described anion leak mediated by ASCT2 could have some relationships with the cysteine-induced efflux; indeed, the anion leak is measured in the presence of cysteine, added to the assay medium as an inhibitor (Zander et al., 2013). These results reinforce the conclusion that the human ASCT2 cannot be considered a cysteine transporter, but a high affinity glutamine transporter.

### Functional oligomerization of ASCT2

**Figure 4:**
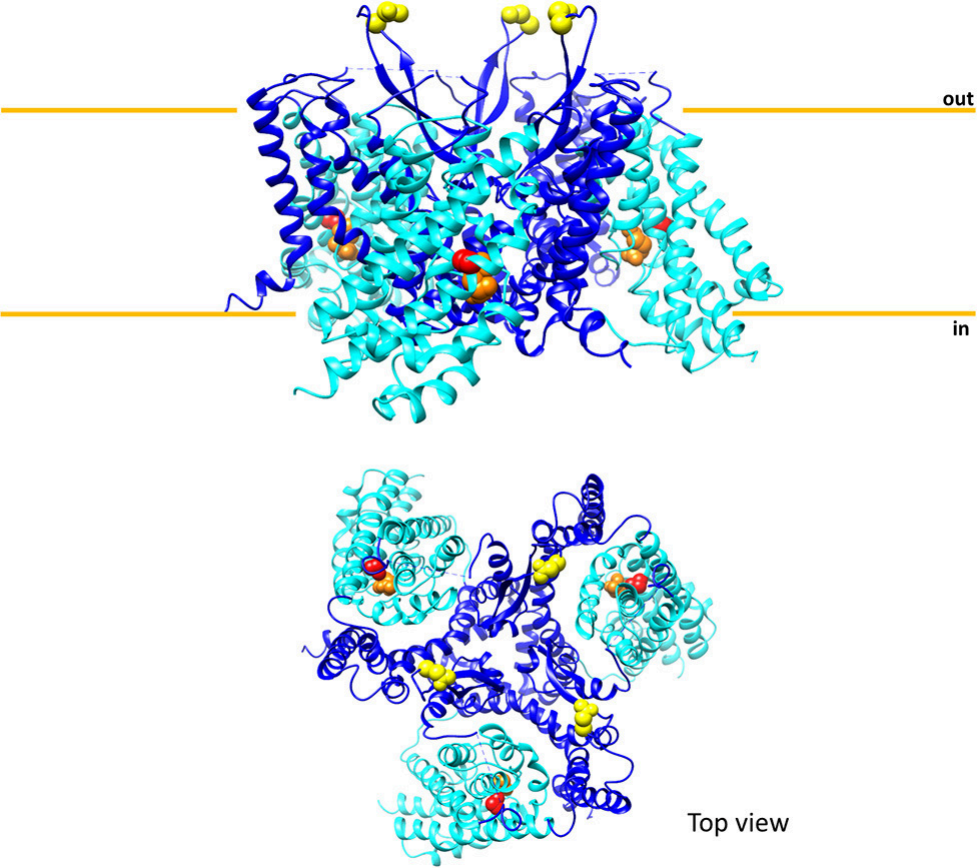
Sketch of the three-dimensional structure of human ASCT2. The above panel shows the overall structure of trimeric human ASCT2 parallel to membrane; each monomer is represented with the fixed domain (blue) and elevator domain (light blue) as in Figure 3. In red, the residue C467 of the substrate binding site with glutamine (orange). N212 residues are depicted in yellow indicating the glycosylation sites; the N163 residues are missing because are not solved in the structure. The membrane is indicated by orange lines. The below panel shows the top view of the same structure. The picture is obtained using Chimera 1.11.2 and the 3D structure coordinates of hASCT2 (PDB: 6GCT) (Garaeva et al., 2018).

Another point, difficult to investigate in intact cells, is the transport mechanism, previously revealed for the rat isoform in proteoliposomes (Oppedisano et al., 2007). The same approach has been used for the human ASCT2: pseudo-bi-substrate kinetic analysis demonstrates that the human transporter gives rise to a random simultaneous mechanism, as well. To fulfill this kinetic mechanism of transport, an oligomeric quaternary structure of ASCT2 is expected in which different monomers work independently, exposing substrate binding sites to the external and internal faces of the protein at the same time (Figure 3). The existence of such a structure was firstly hypothesized on the basis of the 3D structure of bacterial GLTph that works as a trimer (Yernool et al., 2004; Ryan et al., 2009). Therefore, by using biochemical approaches such as mass spectrometry, cross-link and mild-denaturing gel, it has been shown that human ASCT2 forms dimers and trimers which are functional, as assessed by transport assay (Pingitore et al., 2013; Scalise et al., 2014). Later on, the 3D structure of the human EAAT1 (SLC1A3) has been solved showing also an oligomeric conformation, further confirming that this is a feature shared by all members of the SLC1 family (Canul-Tec et al., 2017). This template has been used to build a more refined homology model of human ASCT2 and to study the substrate binding site by site-directed mutagenesis (see section Protein-Protein Interactions, PTM and Structure/Function Relationships) (Scalise et al., 2018b). The trimeric structure of human ASCT2 is definitively demonstrated by the recently solved 3D structure (Garaeva et al., 2018) (Figure 4).

## Protein-protein interactions, PTM and structure/function relationships

One of the most intriguing aspects of cell biology is the regulation of proteins via interactions with other proteins and/or by Post-translational Modifications (PTMs). The interest around these issues relies on the high number of possible combinations; it has to be taken into consideration, indeed, that the same molecule can have more than one interactions and/or modifications in different moments of cell life and that these changes can be transient and/or stable (Venne et al., 2014; Csizmok and Forman-Kay, 2018). This matter is not well-developed for membrane transporters due to the delay in defining their function and regulation. Notwithstanding, in the recent years, rough proteomics data gave some new clues on protein-protein interactions and PTMs. Regarding ASCT2, however, some experimental information is also provided combining *in silico, in vitro*, and *ex vivo* dedicated approaches. Regarding protein-protein interaction, little detailed information is available so far. However, several pieces of evidencefrom *in silico* predictions as well as from immunoprecipitation or double hybrid technologies highlight a sizable number of interactions of ASCT2 with many proteins and also with some viral proteins (https://thebiogrid.org/112401; https://string-db.org/network/9606.ENSP00000444408; https://mint.bio.uniroma2.it/index.php/results-interactions/?id=slc1a5; https://www.ebi.ac.uk/intact/interactors/id:Q15758*).

### Protein-protein interactions of ASCT2

A model of super-complex has been proposed in which ASCT2 interacts with CD147/MCT1 and CD98/LAT1 in order to respond to metabolic regulation by mTOR (Xu and Hemler, 2005). Later on, it has been demonstrated by *in vitro* approaches that human ASCT2 physically interacts with PDZK1. This is a scaffold protein involved in the regulation of a number of SLC members (Kato et al., 2005; Hu et al., 2009). The interaction occurs through a class I PDZ binding domain that represents the most common interaction domain encoded by the human genome (Kim et al., 2012; Scalise et al., 2014). Another PDZ containing protein, referred to as SNX27, binds to ASCT2 increasing its stability in the plasma membrane (Yang et al., 2018). Interestingly, SNX27 is also responsible for the trafficking of GPCRs (G-protein-coupled receptors) and GLUT1 to the plasma membrane (Steinberg et al., 2013). Recently, a paper reported that ASCT2 interacts also with the serotonin transporter (SERT) stimulating the uptake of serotonin in cells (Seyer et al., 2016). This cross talk may have a biological relevance in the homeostasis of neurons. In the frame of cancer biology, a physical interaction between ASCT2 and EGFR has also been proposed that is in good agreement with the regulation of ASCT2 exerted by EGF (see section Regulation of ASCT2 Expression) (Tao et al., 2017).

### Post-translational modifications of ASCT2

#### Role of N-glycosylation: trafficking to plasma membrane and interactions with viruses

The N-glycosylation status of ASCT2 has been investigated being the canonical path through which cells indicate the definitive localization of a protein in the plasma membrane (Li et al., 2004; Haga et al., 2011; Hayashi and Yamashita, 2012). It has been shown that ASCT2 extracted from human cells has an apparent molecular mass higher than that corresponding to the sole polypeptide suggesting the presence of N-glycosyl moieties. Upon treatment with N-glycosidase that removes the N-glycosyl moieties, the ASCT2 apparent molecular mass matches that of the sole polypeptide. The biological significance of N-glycosylation has been evaluated by site-directed mutagenesis, substituting the residues N163 and N212, predicted by bioinformatics to be the target of glycosylation. Figure 4 shows that in the 3D structure recently solved, N212 is exposed to the extracellular environment while N163 is missing because there is a gap in the structure (Garaeva et al., 2018). Substitution of both the asparagine residues with glutamine produces a un-glycosylated protein that is defective in trafficking to the membrane (Console et al., 2015). In term of intrinsic transport activity, parallel assays in intact cells and in proteoliposomes demonstrate that glycosylation/deglycosylation status does not affect the capacity of ASCT2 to catalyze the physiological Na^+^-dependent amino acid antiport, i.e., the glycosyl moiety is not involved in the transport function. This result confirms the suitability of the recombinant human ASCT2 that is not glycosylated, for functional characterization (Pingitore et al., 2013; Console et al., 2015). Very recently, the crystallization study by Garaeva et al. reaches the same conclusion (Garaeva et al., 2018). N-glycosylation of human ASCT2 has been shown also in intact cells by using tunicamycin, a nucleoside antibiotic that blocks the first step of N-glycoprotein synthesis. In this condition, ASCT2 shows an apparent molecular mass corresponding to the sole polypeptide (Polet et al., 2016). In the same work, a link between the glucose metabolism and the extent of N-glycosylation of ASCT2 has been proposed suggesting the existence of a coordinate regulation of glucose and glutamine metabolism (see section Regulation of ASCT2 Expression). A role of N-glycosylation has been proposed regarding the function of human ASCT2 as a viral receptor. It has been firstly hypothesized that glycosyl moieties are responsible for interaction with a group of retroviruses named HERVs. However, from comparison with murine ASCT2 that has not a viral receptor role, it has been shown that the receptor function of the human orthologue relies on an amino acid moiety rather than glycosylation state (Marin et al., 2003). The stretch responsible for the receptor function is the β-sheet exposed toward the extracellular side (Garaeva et al., 2018), which is the most divergent moiety between the human and the murine orthologs (Pochini et al., 2014).

#### Proteomics of post-translational modifications

Another common PTM is the phosphorylation of serine, threonine and tyrosine residues (Aebersold et al., 2018). Phosphorylation is normally linked to the peculiar stage(s) of the protein function being either an activation or an inhibition signal. Regarding ASCT2, a very first paper shows that the uptake of radiolabeled serine is stimulated by SGK1, 3 and PKB kinase activities in *X. laevis* oocytes expressing the human isoform. It has to be stressed that in this first study, a phosphorylation motif in the ASCT2 sequence has not been identified nor hypothesized. Therefore, the positive effect of kinases activity on Vmax of ASCT2, without affecting its Km, is ascribed to a phosphorylation of ASCT2 in a non-canonical site or to the effect of another unknown protein undergoing phosphorylation and modulating in turn ASCT2 activity (Palmada et al., 2005). A number of ASCT2 PTMs can be inferred from rough data of proteomics, summarized in Table 1. In particular, 9 serine residues, 2 threonine residues, and 2 tyrosine residues are reported as phosphorylation sites and it is worth of note that Ser 535 is part of a phosphorylation motif recognized by SGK3 that was not identified in the very first report. Moreover, 7 lysine residues are sites of ubiquitination, regulating ASCT2 stability in cells. Finally, 1 lysine residue and 1 arginine residue are acetyl and monomethyl-derivatives, respectively (Table 1). It is important to highlight that, elucidation of the biological significance of these PTMs is far from being understood.

**Table 1:** Residues of human ASCT2 with PTM according to Phosphosite https://www.phosphosite.org/proteinAction.action?id=7204&showAllSites=true (Hornbeck et al., 2015).

**PTM type**	**Residues of PTM (human ASCT2)**
Serine phosphorylation	9, 27, 183, 194, 198, 493, 503, 535, 539
Threonine phosphorylation	494, 532
Tyrosine phosphorylation	38, 524
Lysine ubiquitination	10, 178, 247, 372, 502, 522, 537
Lysine acetylation	537
Arginine monomethylation	525

### Redox regulation of ASCT2 transport activity

**Figure 5:**
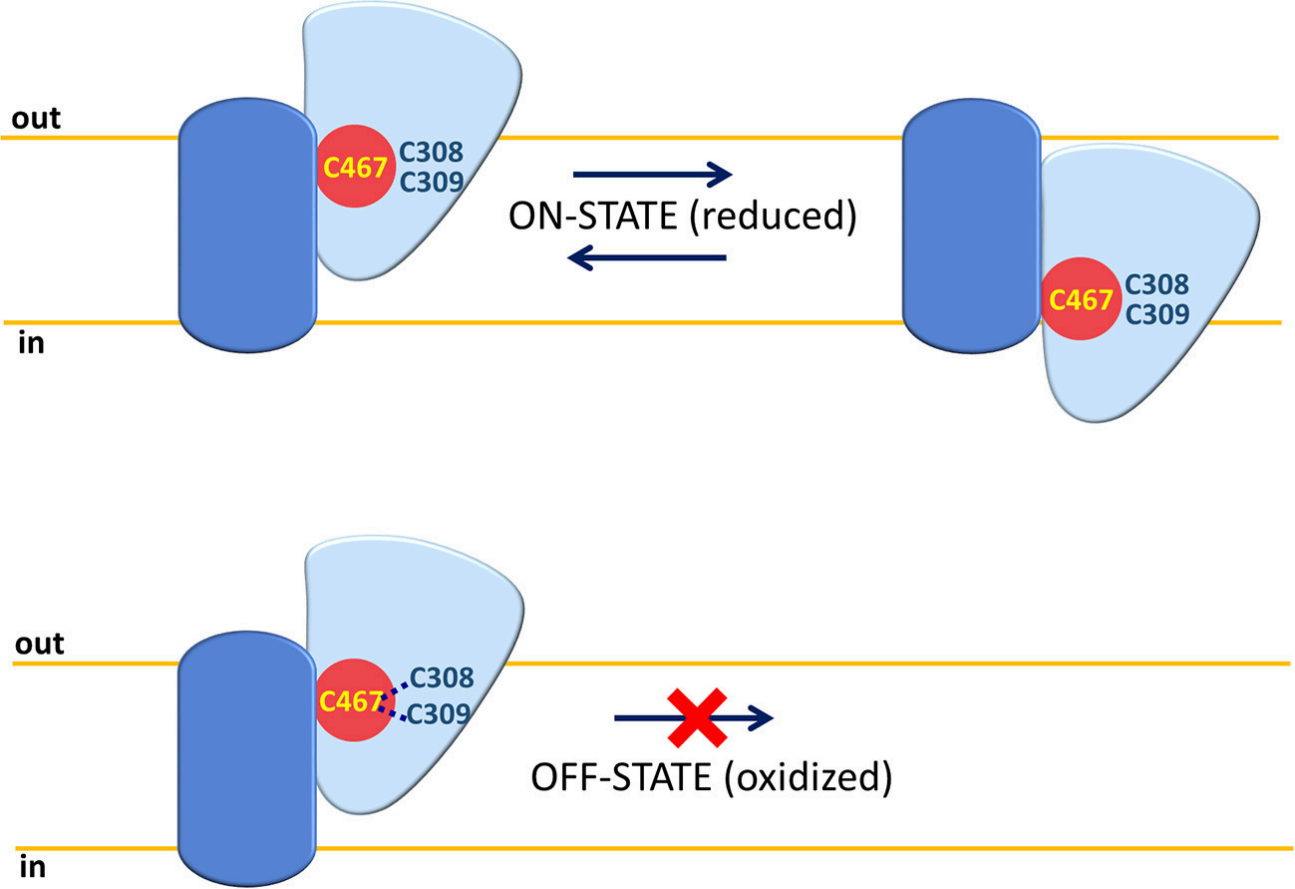
ON-OFF Regulation of human ASCT2. A single monomer of ASCT2 is depicted with the same shapes and colors of Figure 3 (fix domain, blue; mobile domain, light blue; amino acid substrate binding site red). C467 is represented in yellow and C308 or C309 residues close to C467 are depicted in blue. In “ON” state, the Cys residues are in a reduced (SH) form and the mobile part can slide across the membrane for substrate translocation. In “OFF” state, the C467 is oxidized by forming an S-S bond with C308 or C309; the mobility of the sliding moiety is impaired by the disulfide with consequent inactivation of the ASCT2 transport cycle.

Among protein modifications, it has to be considered that cysteine is a versatile amino acid that can give rise to a different kind of PTMs. These are involved, besides other functions, in responding to the redox status of the cell (Giangregorio et al., 2013, 2016; Dong Z. et al., 2017; Mills et al., 2018). In this respect, studies conducted using human ASCT2 reconstituted in proteoliposomes show that the protein is sensitive to both physiological and non-physiological compounds, which can influence the redox status of vicinal cysteine couples or can directly react with such a residue. Both physiological and not physiological reducing agents are able to stimulate Vmax of transport without affecting affinity toward substrates. On the contrary, oxidizing agents (S-S forming) impair protein function. These data indicate that functional ASCT2 has a preference toward reduced cysteine residues, i.e., free thiolic state rather than disulfide form (Scalise et al., 2018b). To gain further insights in the role of the cysteine residues of the human ASCT2, site-directed mutagenesis was performed to construct 8 Cys-Ala mutants that were over-expressed in *P. pastoris*, purified and reconstituted in proteoliposomes for functional assays. The collected data highlight that seven out of eight cysteine residues are not crucial for transport activity and for responsiveness to SH reagents. The sole C467, located in a core region, is involved in substrate binding and translocation as well as in responsiveness toward SH reagents such as GSH, H_2_S, and GSNO used at physiological concentrations. The same residue is also responsive to the non-physiological DTE and to mercury compounds. The C467A mutant, in fact, lost sensitivity to both reducing and oxidizing agents. These results indicate an “ON/OFF” regulation of ASCT2 relying on C467. Physiologically, the cysteine residue could be implied in the formation of S-S with the vicinal C308 or C309 leading to interconversion between the active reduced (SH state) protein to the inactive oxidized (S-S state) protein (Figure 5).

### Functional aspects of human ASCT2

The results derived from site-directed mutagenesis coupled to transport assays can be interpreted in terms of structure/function relationships using a suitable homology model built on the basis of the human EAAT1 (SLC1A1, PDB 5LLU). In this model, it has been suggested the putative binding site for glutamine given that C467 lies on the same position of R457 of EAAT1 and T459 of ASCT1, which are crucial for interaction with glutamate and neutral amino acids, respectively (Scopelliti et al., 2013; Grewer et al., 2014; Colas et al., 2015; Canul-Tec et al., 2017). Moreover, C467 is surrounded by residues (D464, S351, N471, and S343) overlapping those responsible for glutamate binding in human EAAT1 (Hu et al., 2009; Canul-Tec et al., 2017). Besides C467, another cysteine residue, namely C363, revealed crucial for protein stability. Indeed, the substitution C363A does not change the reactivity toward SH reagents but impairs the efficiency of the yeast *P. pastoris* in producing the mutant protein (Scalise et al., 2018b). The structure/function relationships data derived from *in silico* homology models and experimental data on Cys-Ala mutants allowed us to infer some more general conclusions on ASCT2 structure and transport mechanism. Previous studies on recombinant human ASCT2, show that the substrate transfer from one membrane side to the other occurs according to the elevator mechanism, firstly described for the bacterial homolog GLTph (Lee et al., 2013; Akyuz et al., 2015) (Figures 3, 4). Based on this model, the elevator is constituted by a domain containing two transmembrane hairpins called HP1 and HP2. This mobile part is anchored to a fixed domain of the protein embedded in the membrane; the sliding of elevator domain enables substrate translocation (Figures 3, 4). The mobile portion harbors 5 out of the 8 cysteines of human ASCT2 including C467 and C363. Interestingly, C363 is the sole cysteine residue conserved among all the human SLC1 members and is located in the hairpin called HP1b. Importantly, the recent 3D structure of human ASCT2 confirmed the results reported above (Garaeva et al., 2018) (Figure 4) representing a fundamental pre-requisite to inform important emerging studies in ASCT2 biology, i.e., interactions with drugs and xenobiotics (see section Involvement in Human Pathology and Interaction With Drugs).

## Regulation of ASCT2 expression

The regulation of ASCT2 expression has been under investigation since 2004. Notwithstanding the efforts for elucidating the regulatory mechanisms, an integrated view of the pathways is far from being clear. One of the problems arises from the used models: the majority of the data are collected in cancer or immortalized cell lines. These have the advantage of being suitable for gaining reproducible information. However, owing to the metabolic remodeling of cancer cells, the described molecular mechanisms are plausibly different from those occurring in normal tissues. One of the first reports on ASCT2 regulation has been obtained using human hepatoma cells: in this model, it has been proposed that glutamine availability induces the formation of FXR/RXR dimer able to bind to IR-1 repeat in the putative promoter region of ASCT2. This event leads to the activation of ASCT2 expression (Bungard and McGivan, 2004, 2005). Interestingly, glutamine seems to regulate also the expression of FXR, together with glucose, giving rise to a feedback mechanism (Bungard and McGivan, 2005).

### Regulation in cancer

Regulation of ASCT2 by transcription factors involved in cancers and glutamine metabolism has been reported in agreement with the role of this membrane transporter in supplying cells with amino acids responsible for metabolism and signaling pathways. In particular, the down regulation of tumor suppressor pRb, normally observed in cancer, leads to over-expression of ASCT2 through E2F-3 transcription factor (Reynolds et al., 2014). Stability of ASCT2 in the plasma membrane of enterocytes, derived from Caco2 cells, is increased through activation of MAPK and PI3K pathways (Avissar et al., 2008). As a matter of history, in the paper by Avissar et colleagues, ASCT2 and ATB^0^ are reported as two different proteins even though it was already demonstrated that are encoded by the same gene (see section Transport Properties). In breast cancer HR-positive, c-Myc regulates different proteins involved in glutamine metabolism including ASCT2 and glutaminase (Gao et al., 2009; Chen et al., 2015). In the same cells, as well as in a mouse model, a molecular mechanism of activation of ASCT2 expression via the tyrosine kinase receptor EphA2 has been recently reported (Edwards et al., 2017). The importance of ASCT2 in breast cancers is demonstrated also by the activation of ubiquitin ligase RNF5, ascribed to chemotherapy-induced ER stress, that leads to degradation of glutamine transporters including ASCT2 (Jeon et al., 2015; Moses and Neckers, 2015). A link with c-Myc is also reported in hepatocarcinoma cells where, levels of c-Myc, directly correlate with those of mTORC1 activation as well as of ASCT2 (Liu P. et al., 2017). Regulation by micro RNA has been also reported: ASCT2 is downstream of the miRNA-137 in an inversely correlated manner: methyl-CpG-binding protein 2 (MeCP2) and methyltransferases (DNMTs) are responsible for methylation of promoter region of miRNA-137 with its consequent inhibition; conversely, ASCT2 is activated together with glutamine metabolism (Dong J. et al., 2017).

#### Role of mTOR in ASCT2 regulation

As well-acknowledged, the master regulator of sensing nutrient viability in cells is the protein mTOR responsible for modulation of a sizable number of pathways. Based on these premises, it is not a surprise that behind the above-described mechanisms of ASCT2 expression modulation, mTOR may play a role of director as already suggested in the very first report dealing with ASCT2, LAT1 and cancer (Fuchs and Bode, 2005). In this respect, (Fuchs et al., 2007) suggested that the well-known mTOR inhibitor, rapamycin, causes a reduction of ASCT2 expression, that in turn regulates mTOR activity indicating the existence of a reciprocal effect (Fuchs et al., 2007). In 2009, in agreement with the above-mentioned studies, it has been suggested that transport activity mediated by ASCT2 is directly responsible for mTOR induced autophagy in cervical cancer thanks to synergistic action with another plasma membrane transporter for neutral amino acids, i.e., LAT1 (Nicklin et al., 2009). This idea of cross-talk between the two transporters linked to mTOR activity and vice versa endured for some years. However, some recent works showed that even though ASCT2 transport activity is indispensable for tumor growth, it is not always linked to mTOR activation/regulation (Bröer et al., 2016; Cormerais et al., 2018). In fact, very recently, the same group that initially proposed the mutual link between ASCT2 expression and the mTOR pathway likewise reported that mTOR signaling is unaffected in epithelial and mesenchymal human liver cancer cell ASCT2 knockout cells (Bothwell et al., 2018).

#### Cross-talk between glutamine and glucose metabolism

Over the years, the relationship between glutamine and glucose metabolism became more and more evident in either physiological or pathological conditions (Ganapathy et al., 2009). In this respect, mTOR received again much attention since it is at the crossroad between different metabolic pathways. Some experimental findings correlate with this integrated view. In cervical cancer cell lines, lactate which is a product of either glutamine or glucose metabolism stimulates ASCT2 expression (Pérez-Escuredo et al., 2016). Moreover, the cytokine IL4 and activation of its receptor IL4R, are responsible for concerted regulation of GLUT1 and ASCT2 expression in breast cancer (Venmar et al., 2015). The same applies to the transcription factor PPARdelta that has been shown to promote tumor progression of colorectal cancer, breast cancer and cervical cancer by activating ASCT2 and GLUT1 expression (Zhang et al., 2017). Interestingly, a recent paper showed that inhibition of glucose metabolism leads to a reduction of ASCT2 glycosylation (see section Protein-protein Interactions, PTM and Structure/Function Relationships) in agreement with a refined concerted regulation of the two metabolic pathways (Polet et al., 2016).

### Regulation of ASCT2 expression in non-cancer tissues

Even if ASCT2 regulation has been studied almost exclusively in cancer cells, few studies have been published dealing with regulation of this protein in non-cancer tissues, as well. In particular, in oocytes injected with ASCT2 RNA, it has been shown that the kinases SGK1, 3 and PKB can increase ASCT2 abundance in plasma membrane even though the mechanism was not known at that time (Palmada et al., 2005) (see section Protein-protein Interactions, PTM and Structure/Function Relationships). It is important to highlight that these kinases are linked to insulin signaling and EGF pathway correlating well with the effect of EGF on ASCT2. Another study shows that glutamine enters through ASCT2 in porcine enterocytes, enhancing growth via both mTOR and AMP signaling pathways (Yi et al., 2015). In the rat intestine, leptin inhibits ASCT2 expression; interestingly the satiety hormone is involved in regulating the expression of several membrane transporters responsible for nutrient distribution (Ducroc et al., 2010). A positive regulation by aldosterone is also reported for rat ASCT2 in enterocytes overlapping that of LAT1, LAT2, and CD98, being in the frame of a concerted action toward nutrient transporters (Amaral et al., 2008). In mouse mammary gland, the ASCT2 expression is linked to GPCR that acts as mTOR dependent amino acid sensor and modulates the production of proteins in milk. Therefore, ASCT2 may be proposed as a molecular target for modulating milk protein synthesis (Liu J. et al., 2017).

Finally, a link between mTOR-dependent ASCT2 expression and infection by *Salmonella enterica* has been described. Upon infection of human enterocytes by this bacterium, amino acid starvation occurs that, at a later stage of infection, stimulates recruitment of mTOR. The activity of this kinase requires ASCT2 of host cells, together with LAT1 (Tattoli et al., 2012). This mechanism of crosstalk between host and pathogen is intriguing and needs further studies to be fully understood. In fact, several clues in scientific literature suggest that ASCT2 may have a role also in response to pathogens: (i) it can be also exploited as Trojan horse by some viruses to enter cells and (ii) it plays an important function in damaged cells when they start recovering. However, this puzzling scenario does not have yet the common “fil rouge” linking all pieces. Altogether, the knowledge of ASCT2 expression regulation is intricate and, at the present stage, confusing. A summary of the above information is reported in Table 2.

**Table 2:** Regulation of ASCT2.

**Tissue**	**Regulatory factor**	**Effect**	**References**
Hepatoma cells	Glutamine through FXR/RXR	Upregulation	Bungard and McGivan, 2004, 2005
	mTOR inhibition by rapamycin	Downregulation	Fuchs et al., 2007
	c-Myc	Upregulation	Liu P. et al., 2017
Breast cancer HR positive	c-Myc, EphA2	Upregulation	Gao et al., 2009; Chen et al., 2015
	Ubiquitine ligase	Degradation	Jeon et al., 2015; Moses and Neckers, 2015
	IL4 and IL4R	Upregulation	Venmar et al., 2015
	PPAR delta	Upregulation	Zhang et al., 2017
Colon cancer	Repression of miRNA-137	Upregulation	Dong J. et al., 2017
	MAPK-PI3K	Increased stability	Avissar et al., 2008
	PPAR delta	Upregulation	Zhang et al., 2017
Cervical cancer	Lactate	Upregulation	Pérez-Escuredo et al., 2016
	PPAR delta	Upregulation	Zhang et al., 2017
Leukemia cells	Inhibition of glucose metabolism	Downregulation	Polet et al., 2016
MEF	Down-regulation of pRb	Upregulation	Reynolds et al., 2014
*Oocytes expressing hASCT2*	SGK1, 3 and PKB	Increased abundance in plasma membrane	Palmada et al., 2005
*Porcine enterocytes*	mTOR and AMP	Upregulation	Yi et al., 2015
*Rat Intestine*	Leptin	Downregulation	Ducroc et al., 2010
*Rat enterocytes*	Aldosterone	Upregulation	Amaral et al., 2008
*Mouse mammary gland*	GPCR	Upregulation	Liu J. et al., 2017
*Infection by Salmonella enterica*	mTOR	Upregulation	Tattoli et al., 2012

*In plain text, studies conducted in cancer cells, in italics studies conducted in normal cells*.

## ASCT2 and glutamine in cell metabolism

**Figure 6:**
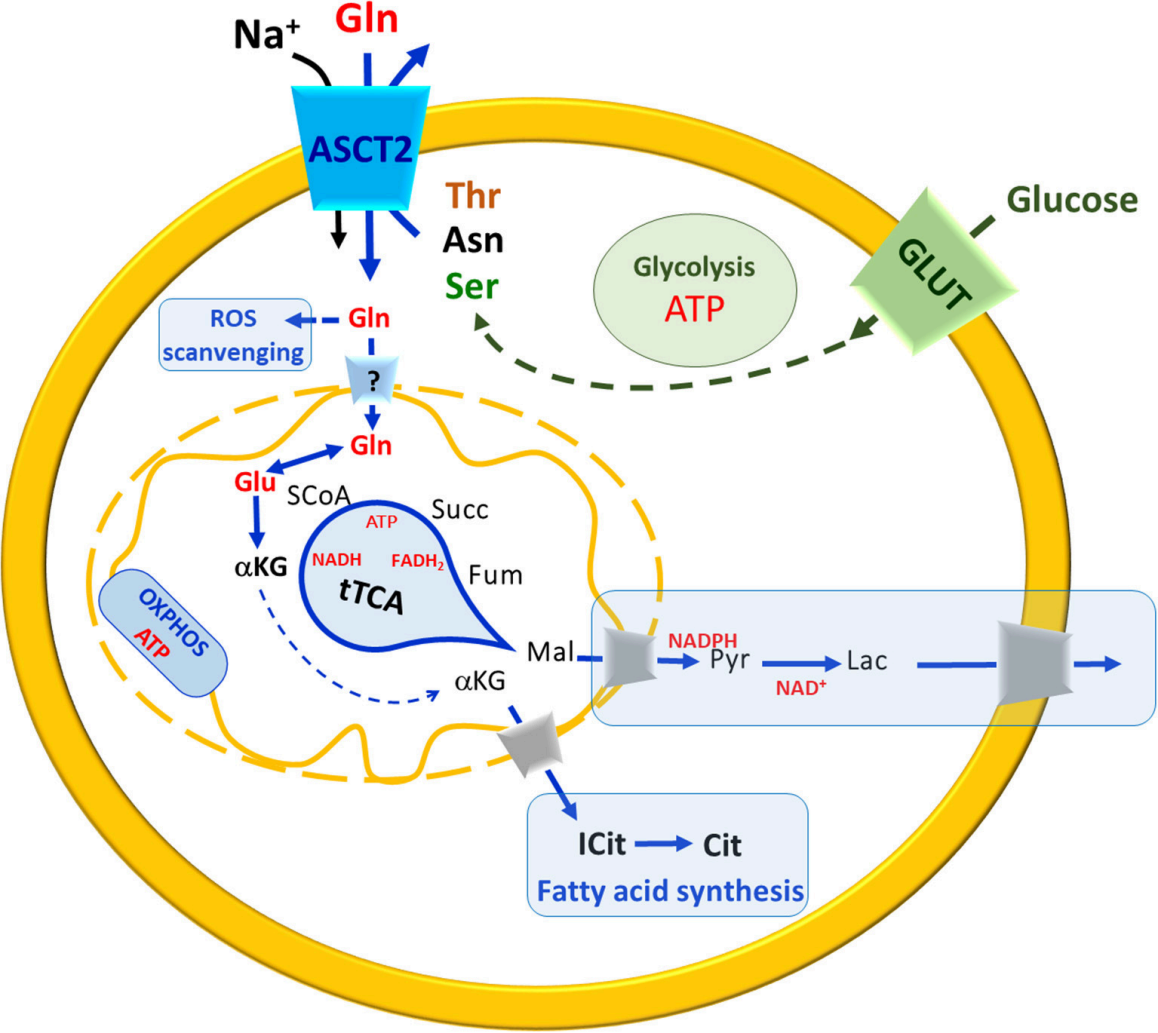
A brief Summary of metabolic changes occurring in highly proliferative cells. In the scheme, uptake of glutamine (Gln, red) and glucose (green) occur mainly via membrane ASCT2 in a sodium-dependent reaction and via GLUT, respectively. The pathways are indicated in blue arrows (when related to Glutamine) in green (when related to glucose). Solid arrows indicate a single reaction(s), while dotted a multistep chain of reactions. The uptake of Gln across the plasma membrane is guaranteed by exchange with smaller amino acids such as threonine (Thr), asparagine (Asn) or serine (Ser). Carbon skeleton of asparagine (black) and serine (green) may derive from malate (black) and glucose (green), respectively. In the cytosol, Gln is used to produce glutathione for ROS scavenging. The entry of Gln in mitochondria is still questionable (“?” In the transporter across the inner mitochondrial membrane). In mitochondria, Gln is converted to Glu that enters the truncated TCA (tTCA) as α-ketoglutarate (αKG) which is also exported to the cytosol for fatty acid synthesis (boxed in light blue). The substrates of tTCA are indicated in black, malate (Mal) reaches cytosol where undergoes a chain of reactions (boxed in light blue) to restore NADPH and NAD^+^ pools. OXPHOS is also depicted in the inner mitochondrial membrane. ATP and reducing equivalent molecules produced by Gln and glucose metabolism are indicated in red.

In recent years, the glutamine metabolism has received great attention. Indeed, it is now evident that approaches as single gene silencing, over-expression, as well as, omics, *per se*, cannot give comprehensive information on the role of a cell molecular component if it is not studied in the context of a metabolic network. The major pathways connected with ASCT2 are those underlying the production/utilization of glutamine. This is a “conditionally essential” amino acid characterized by relatively high plasmatic and intracellular concentrations (Cynober, 2002; Newsholme et al., 2003; Curi et al., 2005), which are finely controlled to fulfill the needs of different body districts (see Introduction). In general, cells which undergo high proliferative rate, have an increased need for glutamine that is used as a source of carbon, by reductive carboxylation, together with glucose; this condition occurs in both physiological and pathological situations leading to a metabolic remodeling characterized, among other modifications, by a truncated form of the mitochondrial tricarboxylic acid cycle (tTCA) (Alberghina and Gaglio, 2014). In particular, the utilization of glutamine carbon skeleton for energy production is based on this “semi-linearized” cycle, in which the glutamine skeleton enters the cycle as α-ketoglutarate, but escapes as malate at about half the cycle and reaches the cytosol where it is used in other pathways for NAPDH and pyruvate production, which are in turn involved in anabolic reactions and in anaerobic glycolysis (Figure 6). As an example, α-Ketoglutarate derived from glutamine skeleton can be a substrate of a cytosolic isoform of IDH (Ricoult et al., 2016). In this non-conventional reaction, isocitrate and citrate are produced for fatty acid synthesis (Mullen et al., 2014). In this metabolic switch, mitochondrial oxphos is not fully functional and ATP is mainly produced at substrate level also in mitochondria by the succinyl-CoA synthetase reaction, with the release of one glutamine carbon atom as CO_2_. One recent hypothesis is that this chain of reactions is sustained by the antiport transport mechanism of ASCT2 that allows net accumulation of glutamine in exchange with smaller substrates such as asparagine and serine. The following experimental evidence supports the hypothesis: (i) asparagine and serine concentrations in cancer cells are higher than in normal ones (Bi and Henry, 2017); (ii) the two amino acids may derive from malate and from glucose, respectively, representing a further proof of a concerted network between glutamine and glucose metabolism for ATP production.

### Glutamine and ROS scavenging

Besides energy production, glutamine is also involved in regulation of ROS scavenging that represents another cross road for cancer cells that are under ROS stress and enhanced glutathione (Glutamate-Glycine-Cysteine-GSH) synthesis (Newsholme et al., 2003; Reynolds et al., 2014). Interestingly, the concept that cancer cells have a controversial behavior toward ROS has been proposed at the very beginning of these studies by Ganapathy and coworkers suggesting that ROS, on the one hand, lead to DNA damages causing DNA mutation and hence cancer malignancy; on the other hand, ROS can be dangerous for cancer itself, because DNA mutations are random and hence potentially lethal even for cancer cells (Ganapathy et al., 2009). Thus, a balance is required to allow survival; this idea has been later developed and demonstrated to involve other transporters for amino acids such as LAT1 and xTC (SLC7A11) (Jiang et al., 2015; Cormerais et al., 2016, 2018). The completion of the puzzle is hampered by different question marks, the most important of which is: does glutamine enter the mitochondrial matrix or is converted to glutamate before crossing the inner membrane? Indirect biochemical data and *in silico* analyses suggest the existence of a glutamine transporter whose molecular identity is however still undefined (Pochini et al., 2014; Scalise et al., 2017) (Figure 6).

### Glutamine in stem cells

An intriguing aspect is that activated immune cells and stem cells increase the need for glucose and glutamine (Oburoglu et al., 2014; Poffenberger and Jones, 2014; Ren et al., 2017). Stem cells are, indeed, characterized by high proliferative rate and metabolic demands; in this respect, they resemble cancer cells and show a Warburg-like metabolism (Agathocleous and Harris, 2013). Moreover, signaling pathways that control their proliferation are also involved in metabolism control. These features are shared with cancer that is indeed considered a disease of differentiation in which cells progressively reach an undifferentiated state to be more proliferative (Agathocleous and Harris, 2013). A recent paper showed that glucose and glutamine are critical for biosynthetic reactions in hematopoietic stem cells. Interestingly, in these cells, ASCT2 and GLUT1 are responsible for uptake of glutamine and glucose, respectively (Oburoglu et al., 2014). It has to be taken into consideration that the phenomenon of metabolic flexibility has been mainly studied in the context of cancer, so far. It remains to be determined whether such pathways also operate in non-cancerous cells.

## Involvement in human pathology and interaction with drugs

As highlighted in section ASCT2 Gene Expression, ASCT2 is involved in several human cancers thus being an important target for drugs. However, besides cancer, ASCT2 expression/function is altered also in other pathological conditions such as IUGR (IntraUterine Growth Restriction) (Aiko et al., 2014), ALS (Amyotrophic Lateral Sclerosis) (Lee et al., 2017), and schizophrenia (Maucler et al., 2013). Being a plasma membrane transporter, ASCT2 could also be either a first level drug target or a relevant player in drug disposition, similarly to LAT1 which is an acknowledged transporter of several amino acid-like drugs (del Amo et al., 2008; Fotiadis et al., 2013; Scalise et al., 2018a; Singh and Ecker, 2018). In spite of such evidence, the scientific community, the scientific community still did not include ASCT2, or any other transporter for amino acids, in the list of the International Transporter Consortium for drug-transporter interactions (Giacomini and Huang, 2013).

### Competitive inhibitors of ASCT2

Regarding anti-cancer therapy, in the last 10 years, the research in pharmacology employed great efforts in designing new molecules or in finding serendipitous effects with old drugs unrelated to cancer. In the case of ASCT2, studies have been conducted simultaneously on murine and human isoforms even if, as mentioned above, the molecular and structural differences existing between these proteins may be the principal reasons of the unsuccessful design of new anticancer therapies. The first studies on ASCT2 have been conducted on the rat isoform using substrate mimicking drugs and measuring effects on transport activity as Na^+^–dependent alanine and glutamine induced currents. In these conditions, two molecules, i.e., benzylserine and benzylcysteine, have been identified as specific competitive inhibitors. In particular, the compounds are able to block the current associated with the Na^+^–dependent transport of amino acids. Already in this first report, it is stated that these inhibitors are not suitable as such but they are conceived, as “proof of principle,” that inhibitor design based on substrate analogs is possible (Grewer and Grabsch, 2004). Other molecules have been designed on the basis of glutamine structure moving from the premise that glutamine is the highest affinity substrate and that, lowering the pKa of the amide (NH group), would increase binding affinity to the ASCT2 transporter. Therefore, a p-nitrophenyl analog has been identified as a good inhibitor of rat ASCT2 with an IC50 of 500 μM (Esslinger et al., 2005). In another study, a further refined molecular docking of rat ASCT2 is used with the idea of developing serine-based inhibitors as potential ligands, either transported substrates or blockers. The results are collected measuring, also in this case, electrical currents. In this experimental tool, new compounds with 3-fold higher affinity, with respect to glutamine analogs, are proposed. The kinetics of inhibition is competitive, correlating with the nature of substrate analogs (Albers et al., 2012).

### Covalent inhibitors of ASCT2

Simultaneously, using rat ASCT2 reconstituted in proteoliposomes, the reactivity of the protein toward metals, such as Hg and its organic derivatives, has been evaluated as inhibition of radiolabeled glutamine uptake. This interaction is probably due to the presence of a metal binging motif (CXXC) in the rat sequence (Oppedisano et al., 2010). It is important to remark that, even though the human ASCT2 lost the CXXC motif, it retains a strong reactivity toward Hg derivatives that form bonds with cysteine residues of the protein (Pingitore et al., 2013; Scalise et al., 2018b). The studies on cysteine reactivity put forth the possibility of exploiting them to design potent and specific inhibitors able to irreversibly block ASCT2 activity. This approach would have the advantage of high specificity and high potency, avoiding the problems connected with substrate analogs that can be displaced from the binding site by endogenous amino acids whose concentrations may increase under some conditions with special reference to glutamine (Scalise et al., 2017). This is much relevant in the context of amino acid transporters because the same protein often recognizes more than one amino acid as substrate (Pochini et al., 2014). A library of fifty molecules characterized by a dithiazole moiety but differing in electronic, lipophilic and steric properties of substituents, has been tested on rat ASCT2 reconstituted in proteoliposomes. Among these, six compounds have been identified as potent inhibitors with IC50 ranging from 3 to 30 μM and with the capacity of forming covalent bonds with SH residues of the protein. Computational analysis suggests that these molecules are able to interact with the CXXC motif that is presumably not located in the substrate binding site (Oppedisano et al., 2012). In 2015, using again rat ASCT2 new molecules have been designed, which are able to activate or inhibit transport activity, measured as anion current or as radiolabeled glutamine uptake in intact cells (Colas et al., 2015). In this paper, some structural insights into ASCT2 substrate binding site were proposed, thanks to the refined 3D structure of GLTph available at that time. Recently, another paper has been published revealing some hints on substrate binding site of rat ASCT2 (Scopelliti et al., 2018) with results similar to those obtained with human ASCT2 (Garaeva et al., 2018; Scalise et al., 2018b).

### Proline derivatives as a scaffold for new ASCT2-targeting drugs

Five activators and two inhibitors are described with the intriguing findings of a proline derivative able to activate and another to inhibit transport mediated by rat ASCT2. This result represents a novelty because proline is not a substrate the protein. The two compounds have different size and chemical properties: the activator is a cis-3-hydroxyproline, it is small and possibly it is transported by rat ASCT2 over-expressed in human HEK293 cells; the inhibitor is characterized by a FluoroBenzyl substituent on the proline (γ-FBP) and has cytotoxic effects on human melanoma cell line (Colas et al., 2015). Moving from these molecules that, even though designed on rat ASCT2, revealed to be effective also in human cells, many studies faced the interaction of human ASCT2 with drugs. In particular, the benzyl proline derivatives constitute the scaffold for designing new and more potent molecules against the human isoform. In this work, a Ki of 3 μM is reported indicating that the combination of computational approach and experimental assays is successful (Singh et al., 2017). More recently, a competitive antagonist of ASCT2 has been identified which reached the preclinical phase: from a previous screening conducted in 2015 (Schulte et al., 2015) on a series of the 2-amino-4-bis (aryloxybenzyl)aminobutanoic acids, a lead emerged referred to as V-9302. The ability of this compound to inhibit ASCT2 transport has been predicted *in silico* and then evaluated *in vitro*. It is interesting to note that in mice harboring a Patient-Derived Xenograft (PDX) tumor, the treatment with V-9302 induces a reduction of tumor size. In both *in vitro* and *in vivo* models, the molecular mechanism of inhibition by V-9302 is linked to decreased mTOR activity, which is consistent with lowered glutamine transport and metabolism. Moreover, V-9302 induces autophagy; combining the drug with an autophagy inhibitor a further decrease in tumor cells viability is observed, indicating that a combined therapeutic strategy may further improve the efficacy. Finally, V-9302 also induces oxidative stress that is lethal for cancer cells that, being highly proliferative, need higher levels of glutathione that in turn, requires glutamine (Schulte et al., 2018). The structure of human ASCT2, recently solved, will certainly open new perspectives in pharmacological studies related to cancer but also to the other pathologies in which ASCT2 is involved.

## Conclusions

The plasma membrane transporter ASCT2 represents a key player in cell biology due to its broad expression and substrate specificity that is adapted to different cell needs. The relevance of this protein in both physiological and pathological conditions is testified by the sizable number of studies that investigate different aspects of the multifaceted function and regulation of ASCT2. In the last 10 years, the efforts in designing new drugs steadily increased due to the recognition that cancer involves derangements in differentiation and glutamine metabolism. Noteworthy, ASCT2 is involved in both these phenomena supporting the idea that targeting this prominent transporter will impair cancer cell viability. However, it has to be taken into consideration that amino acid homeostasis is a complex and intricate network, in which more than one transporter or enzyme handle one or more substrates. Thus, the idea of chemically or genetically blocking only one target cannot be a straightforward strategy applicable to all pathological situations. Recent studies with ASCT2 knockout cells reveal that several compensatory mechanisms compensate for its absence, and that it nonetheless plays a role in tumorigenesis in ways that remain to be elucidated.

## Author contributions

MS contributed in collecting and analyzing bibliography, preparing figures and writing. LP and LC contributed to draw structure figures and writing. ML contributed in drawing figures and tables. CI contributed to write and supervision of all the activities.

### Conflict of interest statement

The authors declare that the research was conducted in the absence of any commercial or financial relationships that could be construed as a potential conflict of interest.

## Abbreviations

mTOR: mammalian Target of Rapamicyn
GCN2: general control non-derepressible 2
TCA: TriCarboxylicAcid cycle
tTCA: truncated TriCarboxylicAcid cycle
NO: nitric oxide
H_2_S: Hydrogen sulfide
SNP: Single Nucleotide Plymorfism
MeAIB: Methylaminoisobutyric acid
BCH, 2-aminobicyclo-(2, 2: 1)-heptane-2-carboxylic acid
ALS: Amyotrophic Lateral Sclerosis
PTM: Post-Translational Modification
GPCR, G-protein-coupled receptors;EGFR: Epidermal Growth Factor Receptor
EGF: Epidermal Growth Factor
FXR7RXR: Farnesoid X Receptor / Retinoid X Receptors
IR-1: Insuline Receptor-1
IL4: InterLeukin4
IL4R: Interlukin 4 Receptor
ROS: Reactive Oxygen Species.
